# Analysis of an Inactive Cyanobactin Biosynthetic Gene Cluster Leads to Discovery of New Natural Products from Strains of the Genus *Microcystis*

**DOI:** 10.1371/journal.pone.0043002

**Published:** 2012-08-27

**Authors:** Niina Leikoski, David P. Fewer, Jouni Jokela, Pirita Alakoski, Matti Wahlsten, Kaarina Sivonen

**Affiliations:** Department of Food and Environmental Sciences, University of Helsinki, Helsinki, Finland; Laurentian University, Canada

## Abstract

Cyanobactins are cyclic peptides assembled through the cleavage and modification of short precursor proteins. An inactive cyanobactin gene cluster has been described from the genome *Microcystis aeruginosa* NIES843. Here we report the discovery of active counterparts in strains of the genus *Microcystis* guided by this silent cyanobactin gene cluster. The end products of the gene clusters were structurally diverse cyclic peptides, which we named piricyclamides. Some of the piricyclamides consisted solely of proteinogenic amino acids while others contained disulfide bridges and some were prenylated or geranylated. The piricyclamide gene clusters encoded between 1 and 4 precursor genes. They encoded highly diverse core peptides ranging in length from 7–17 amino acids with just a single conserved amino acid. Heterologous expression of the *pir* gene cluster from *Microcystis aeruginosa* PCC7005 in *Escherichia coli* confirmed that this gene cluster is responsible for the biosynthesis of piricyclamides. Chemical analysis demonstrated that *Microcystis* strains could produce an array of piricyclamides some of which are geranylated or prenylated. The genetic diversity of piricyclamides in a bloom sample was explored and 19 different piricyclamide precursor genes were found. This study provides evidence for a stunning array of piricyclamides in *Microcystis*, a worldwide occurring bloom forming cyanobacteria.

## Introduction

Cyanobactins are small cyclic peptides with interesting pharmaceutical properties including antimicrobial activity against human pathogens [1]–[4]. Cyanobactins are true ribosomal gene products and made through the enzymatic modification of short precursor peptides [4]–[5]. Cyanobactin biosynthetic gene clusters encode two proteases responsible for the cleavage and cyclization of one or more precursor peptides [4]–[7]. The heterocyclization of threonines, serines and cysteines is a common post-translational modification in cyanobactin biosynthesis [8]–[9]. The heterocyclization of threonines, serines and cysteines to form oxazoles and thiazoles as well as the oxidation to oxazolines and thiazolines has been described in detail [8]–[9]. Disulfide bridge formation and prenylation or geranylation or specific amino acids have also been reported [4], [7], [10], [11], [12]. Prenylation occurs as a last step process after the heterocyclization, cleavage and macrocyclization of the precursor peptide to the final cyclic peptide [11].

The cyanobactin biosynthetic pathway is known from a range of cyanobacteria e.g. *Prochloron, Arthrospira platensis, Planktothrix agardhii, Oscillatoria, Nostoc* and *Anabaena* [7], [10]–[15]. *Microcystis* strains produce microcyclamides, which are hexapeptides with varying amino acid content and which carry heterocyclized amino acids at every second position [3], [15], [16]–[18]. Microcyclamides have been shown to have cytotoxic activities against murine leukemia cells, toxic effects on crustacean *Thamnocephalus platyurus* and *Plasmodium falciparum* [3], [16]. A cyanobactin gene cluster with an unknown end-product was reported from the complete genome of *Microcystis aeruginosa* NIES843 [7], [19]. However, this cluster is disrupted by large insertions and rearrangements and appears to be non-functional [7].

Here we report a natural product discovery driven by bioinformatic analysis of an inactive gene cluster. This new family of prenylated and geranylated cyanobactins, which we named piricyclamides, is common in strains of the genus *Microcystis*.

## Results

### Inactive Gene Cluster used to Find a Functional Counterpart

**Figure 1:**
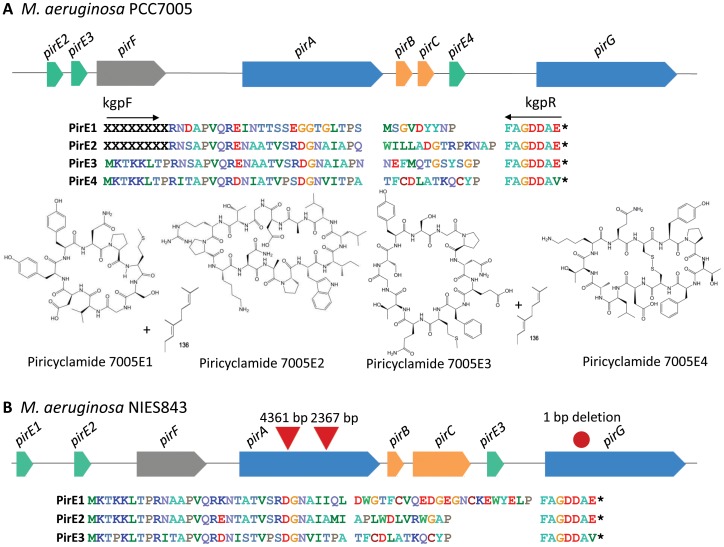
Schematic drawing of the *pir* gene clusters, precursor sequences and structures of piricyclamides. These are found in the genome of *M. aeruginosa* PCC7005 (A) and NIES843 (B). The genes in green are the precursor genes, proteases are blue, prenyltransterase is grey and typical genes for cyanobactin with no predicted function are orange. Piricyclamides produced from PirE1-PirE4 precursors by the *M. aeruginosa* PCC7005 are shown. Above the precursors the site is shown the annealing site of the primers used in the precursor gene amplification. Interruptions of the *pir* genes in *M. aeruginosa* NIES843 are indicated with red triangles and deletion with a red circle at 508 position of *pirG*. The length of the *pir* gene cluster is from *pirE2* to *pirG* 9.4**kb. The entire length of the gene cluster area in *M. aeruginosa* NIES843 including the insertions is from the beginning of *pirE1* to *pirG* is 19.6 kb. The amino acid sequences of the precursors are aligned below the gene cluster.

Bioinformatic analysis demonstrated that the cyanobactin gene cluster from *Microcystis aeruginosa* NIES843 was inactivated by two insertion elements (Figure 1). We screened 74 *Microcystis* strains with PCR and LC-MS to discover active producers of similar cyanobactins. These strains were screened simultaneously by LC-MS based mainly on the fragmentation pattern typical for cyanobactins containing isoprenoid units linked to heteroatoms. The presence of isoprenoid unit (68 Da) is clearly seen in MS and MS^2^ spectra as a neutral loss of 68 and 136 (Figure S1A–C). Potential cyanobactin candidates were found in 10 out of the 74 strains. Simultaneously the piricyclamide precursor genes were amplified by PCR using primers designed to anneal specifically in the N- and C-terminal of all three precursors encoded in the cyanobactin gene cluster in *Microcystis aeruginosa* NIES843. Precursor genes were amplified from 28 out of the 74 strains. Six of these 28 strains produced prenylated cyanobactin candidates detected by LC-MS. These 6 strains for which both precursor genes and candidate cyanobactins could be identified were predicted to contain active piricyclamide gene clusters and were studied further. Clone libraries of the amplified precursors from the 6 *Microcystis* strains, Izancya36, Izancya41, Izancya42, SYKE764, SYKE864 and PCC7005, were constructed and ten clones were sequenced from each library. Each of the 6 strains had between 2 and 4 cyanobactin precursor genes. The end products of the new piricyclamide gene clusters were predicted from the precursor genes (Table 1).

**Table 1:** The piricyclamide core sequences in the studied strains of *Microcystis*.

Piricyclamide core sequence	*Microcystis* strain						
	PCC 7005	Izancya 36	Izancya 41	Izancya 42	SYKE 864	SYKE 764	NIES 843
NEFMQTGSYSGP	Z, G						
TFCDLATKQCYP	Z, SS				X*		X
WILLADGTRPKNAP	Z						
MSGVDYYNP	Z, G						
TLGCMNGTERCLGLP		Z, SS	Z, SS				
DWGTFCVQEDGEGNCKEWYEVP		X	X				
DWGTFCVQEDGEGNCKGWYELP					X		X
GTHLYTITP					Z, P		
APLWDLVRWGAP							X
ILGEGEGWNYNP				Z, P		X	
FAIFLLLP				Z			
YSNVLPP				X			
SQWGWRGLSDP						Z	
GWGTFCVGEDGDGNCEEWYELP						X	

The X indicate the presence of specific core sequence in the strain or bloom sample. The Z indicate that the piricyclamide was detected with LC-MS. Posttranslational modifications in addition to cyclization are indicated; G, geranyl, P, prenyl and SS, disulfide bridge.

* interrupted precursor gene.

### Discovery of Piricyclamides

The mass of the putative cyanobactins detected by LC-MS were used together with the precursor peptide sequences to predict cleavage sites. These predictions were confirmed by derivatization and labeling experiments. *Microcystis* strains were cultivated on ^15^N stable isotope containing medium to verify the piricyclamide structures. The cell extracts were analysed by LC-MS. Increased molecular mass values (MH^+^) of the ^15^N-labelled piricyclamides matched with the values calculated from the amino acid sequences and confirmed the number of nitrogen atoms and the piricyclamide structures (Table S4). Four piricyclamides, WILLADGTRPKNAP, FAIFLLLP, SQWGWRGLSDP and GTHLYTITP, contained only proteinogenic amino acids and no further evidence of post-translational modifications other than macrocyclization was found by LC-MS (Figure S1E, F, K and L, Table 1, Table S4, Table S5).

Some of the predicted piricyclamides, MSGVDYYNP, NEFMQTGSYSGP and TLGCMNGTERCLGLP, contained a methionine residue. These piricyclamides had 16 Da higher masses and which eluted slightly earlier in reversed phase chromatography because they contained methionine sulphoxide, a well-known methionine oxidation product that probably had formed during sample preparation. Methionine sulphoxide specific elimination of CH_3_SOH [20] was detected in the product ion spectra of these piricyclamide variants.

Some of the core regions of the precursor peptides displayed a double cysteine pattern suggesting that they encoded piricyclamides which contain disulfide bridges. The presence of sulphur in piricyclamide TLGCMNGTERCLGLP was shown by cultivation of *Microcystis* Izancya 41 in medium containing stable isotope ^34^S instead of standard isotope ^32^S. ^34^S-labelling increased the mass of piricyclamide TLGCMNGTERCLGLP by 4 Da which, demonstrated the presence of two sulphur atoms in the cyanobactin. Disulfide bridges were subsequently identified in two piricyclamides TFCDLATKQCYP and TLGCMNGTERCLGLP (Table 1). Disulfide bridge structure was verified by reduction and cysteine specific carboxyamidomethylation of the peptide [21]. Derivatization shifted the mass of the protonated piricyclamide ion by 116 Da (from *m/z* 1544 to *m/z* 1660 [MH]^+^) demonstrating the existence of a single disulfide bridge. Further proof was obtained from the product ion spectra of the derivatized piricyclamide TLGCMNGTERCLGLP which showed loss of 91 Da fragment H_2_NC( = O)CH_2_SH specific for carboxyamidomethylated cysteine (Figure S1H).

### Chemical Diversity of Piricyclamides

Piricyclamides MSGVDYYNP and NEFMQTGSYSGP in *M. aeruginosa* PCC7005 (Table 1) were detected only 136 Da larger variants than predicted from the precursor peptide sequence. Loss of 136 Da fragment dominated the product ion spectra of both piricyclamides and the loss of the fragment was seen already in ion source (Figure S1B, C). ^15^N-labelling experiment showed that the 136 Da additional structural unit did not contain nitrogen (Figure S1 I, J). These results strongly indicate that the piricyclamides MSGVDYYNP and NEFMQTGSYSGP contain a heteroatom bound geranyl group. The ILGEGEGWNYNP piricyclamide had otherwise identical properties as the geranylated piricyclamides but was detected only 68 Da larger variant than predictable from the sequence (Figure S1A, G). In this case the piricyclamide contained a heteroatom bound prenyl group.

The GTHLYTITP piricyclamide was detected in an unmodified form as predicted from the precursor sequence of the SYKE864 strain together with two larger peptides (84 Da and 84+68 Da) one of which another contained a prenyl unit bound to a heteroatom (Table S4, Figure S2A–C). ^15^N-labelling revealed equal nitrogen content of these three peptides, which motivated the comparison of the product ion spectra. Assignment of ions showed that several tri- and tetrapeptide ions, which did not contain tyrosine (*m/z* 334, 375, 393, 488), were present in GTHLYTITP, prenylated and nonprenylated peptide 1068 spectra. In contrast tyrosine containing tri- and tetrapeptide ions (*m/z* 360 and 497) were present in GTHLYTITP spectrum were missing or had low intensity in Pr-peptide 1068 and peptide 1068 spectra. 84 Da larger ions (*m/z* 444 and 581) were present in Pr-peptide 1068 and peptide 1068 spectra (Figure S3). The higher mass fragment ions behaved identically (Figure S2). These results suggest that Pr-peptide 1068 and peptide 1068 have otherwise same sequence than piricyclamide GTHLYTITP but instead of tyrosine there is an 84 Da larger amino acid or tyrosine derivative. To further elucidate the structure of the possible tyrosine derivative, peptide 1068 was analysed with MALDI-TOF, which gave *m/z* 1068.546 for the protonated ion. Theoretical mass for protonated piricyclamide GTHLYTITP is *m/z* 984.5149 so the mass for the possible additional group in tyrosine was 84.0311 Da. Two rational carbon, hydrogen and oxygen (^15^N labelling showed lack of nitrogen) containing chemical formulas within mass deviation of 50 mDa fitted to the mass and those were C_4_H_4_O_2_ (Δ +14.4 mDa) and C_5_H_8_O (Δ −22.0 mDa). Latter formula could represent a hydroxyprenyl group described as a structural group in xanthone from a rain forest plant psorospermum cf. molluscum [22].

### Inactive Piricyclamide Gene Clusters

Bioinformatic analysis suggested that the cyanobactin gene cluster from NIES843 is inactive (Figure 1). LC-MS analysis also failed to identify predicted cyanobactins from the cell extract of *M. aeruginosa* NIES843. The *pir* gene cluster of *M. aeruginosa* NIES843 is 19 655 bp from 32581–52235. In the previous analysis the gene cluster was analyzed forward from *pirF* gene [7] while two precursor genes appear also upstream from *pirF* making the gene cluster even longer than previously described. The bioinformatic analysis of the *pir* gene cluster was complicated by many interruptions and short hypothetical proteins as mentioned previously [7]. The *pirG* in *M. aeruginosa* NIES843 has a frameshift mutation at 508 position of MAE_00690. We identified two insertion sequence elements in *pirA* gene of NIES843. IS1 has a length of 4361 bp while IS2 has a length of 2367 bp and both insertion sequences are flanked by terminal inverted repeats. IS1 is flanked by a 404 bp duplicated region which contains 100 bp of non-coding promoter and 300 bp of *pirA*. IS2 is flanked by a three base pair direct repeat. The two insertion sequences encode a transposase, which are found in over 30 IS elements in the genome of NIES843. The entire *pir* gene cluster encodes three precursor genes *pirE1-pirE3*. All three precursor peptides had a very similar sequence except for the core peptide region which encoded the putative cyanobactin product. The gene cluster encodes all the essential genes for cyanobactin gene clusters but it lacks the PatD heterocyclase and the PatG heterocycle oxidizing domain from the C-terminal protease (Table S1).

Genetic rearrangements were also identified in precursor genes from *M. aeruginosa* NIES102 and SYKE864 and one from the natural sample, which would lead to silencing of the precursor gene. A 95 bp MITE element was found in one of the *pirE* precursor genes of SYKE 864. This insertion sequence element was characterized by short terminal inverted repeats. We identified frameshift mutations in precursor genes from NIES102 and a cloned *pirE* precursor from Lake Tuusulanjärvi.

### The *pir* Gene Cluster from PCC7005

**Figure 2:**
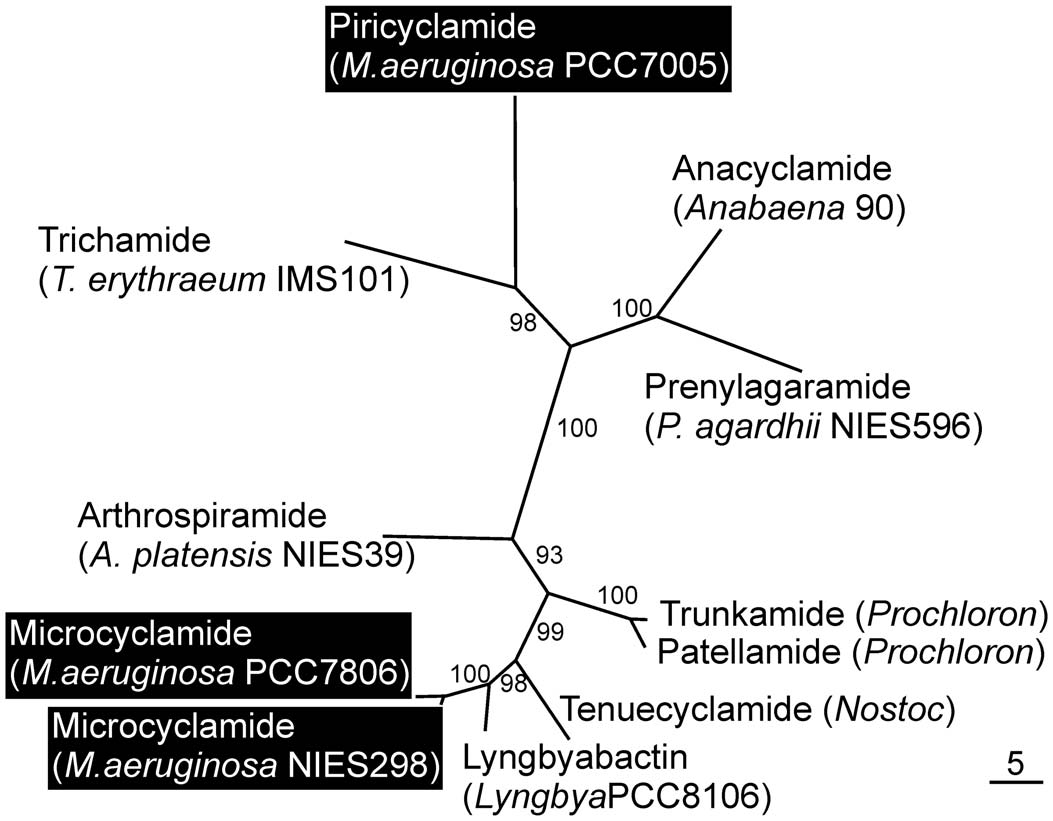
An unrooted maximum likelihood tree constructed from dataset of concatenated proteases (A and G) in cyanobactin producers for which the biosynthetic genes have been described. Bootstrap values are from 1000 ML-bootstrap replicates. The branch lengths are proportional to sequence change. Cyanobactins produced by *Microcystis* strains are highlighted in black.

The *pir* gene cluster sequence in NIES843 was used to design primers to amplify the entire *pir* gene cluster from PCC7005. The core cyanobactin proteins of PCC7005 and NIES843 were almost identical and very similar to anacyclamide, trichamide and prenylagaramide biosynthetic enzymes (Table 2). The *pir* genes are organized in the same order in PCC7005 and NIES843 (Figure 2). However, the size of *pir* gene cluster is ∼9.4 kb in PCC7005 and 19.6 kb in *M. aeruginosa* NIES843. In addition to the three precursors PirE2-PirE4 the PCC7005 *pir* gene cluster encodes two proteases PirA and PirG, putative geranyl transferase PirF, PirB and PirC, which are essential, but the function is unknown (Table 2). The *pirE1* precursor gene was not found in the *pir* gene cluster of PCC7005. The PCC7005 gene cluster was cloned into *E. coli* and sequenced showing that we amplified and cloned the *pir* cluster from the beginning of *pirE3* (Figure 1) onwards instead of entire cluster. LC-MS analysis showed that only piricyclamide TFCDLATKQCYP (*pirE4)* was heterologously expressed in *E. coli.* As the sequencing showed that the amplified part encoded the essential genes and only precursor *pirE4* with its promoter, which is in the middle of the gene cluster. Subsequent attempts allowed the amplification and cloning of the *pir* cluster from the middle of *pirE2* onwards. This part of the PCC7005 *pir* gene cluster was cloned into *E. coli* and sequenced. Again in the LC-MS analysis the same *pirE4* encoded piricyclamide was produced. The fragment cloned into *E. coli* had partial *pirE2* and no promoter for the *pirE3.* Attempts to extend the pir gene cluster to include *pirE1* were unsuccessful.

**Table 2:** The predicted proteins and their proposed functions in the piricyclamide gene cluster of *Microcystis aeruginosa* PCC7005.

Protein	Accession Number	Length (aa)	Predicted function	Blast result	
				Identity (%)	Organism
PirE1	JX129192	48	Precursor	59	*P. agardhii* NIES596
PirE2	AFK79987	53	Precursor	56	*M. aeruginosa* NIES843
PirE3	AFK79988	51	Precursor	56	*M. aeruginosa* NIES843
PirF	AFK79989	296	Prenylation	83	*M. aeruginosa* NIES843
ORF	AFK79990	465	Hypothetical		
ORF	AFK79991	192	Hypothetical		
ORF	AFK79992	227	Hypothetical		
PirA	AFK79993	645	C-terminal protease	67	*T. erythraeum* IMS101
PirB	AFK79994	69	Associated with cyanobactin biosynthesis	99	*M. aeruginosa* NIES843
PirC	AFK79995	73	Associated with cyanobactin biosynthesis	81	*M. aeruginosa* PCC7806
PirE4	AFK79996	51	Precursor	98	*M. aeruginosa* NIES843
ORF	AFK79997	271	Hypothetical		
PirG	AFK79998	689	N-terminal protease, macrocyclaze	96	*M. aeruginosa* NIES843

### Diversity of Piricyclamides in a Natural Sample

The genetic diversity of piricyclamide precursor genes was studied in a bloom sample from Lake Tuusulanjärvi. Bioinformatic analysis of a 16S rRNA clone library constructed from the bloom material suggested the sample to contain mainly *Aphanizomenon* (97%) and *Microcystis* in lower frequencies (3%). The 122 *pirE* precursor genes were sequenced from a library constructed from the bloom material. The amino acid core sequences of the peptide precursors varied extensively (Table 3). The metagenome showed 19 unique piricyclamide precursor genes. Two variants of the precursor core sequence had a double cysteine pattern and therefore the resulting structure would contain a sulphur bridge. The length of hypothetical piricyclamides with disulfide bridges was 10 and 22 amino acids. The remaining 17 piricyclamides had variable core sequence from 7 to 17 amino acids (Table 3). Three of the core sequences of the precursor metagenome were identical to the ones in the strains studied while the remaining 16 putative piricylamides were novel.

**Table 3:** Piricyclamide precursor core sequences found in the bloom sample metagenome of 122 clones from Lake Tuusulanjärvi 1987.

Number of clones	Core sequence	*Microcystis* strain
38	APLWDLVRWGAP	
21	DWGTFCVQEDGEGNCKEWYELP	SYKE864
15	TGNKSGKVTP	
9	QTIGYWKDP	
7	MSGVDYYNP	PCC7005
5	HQSLWAWNGSDP	
4	GSGHPLYP	
4	TVFDYTVP	
4	HQWGWLVGGTDP	
3	TRGCSFCPFP	
2	FITWSWSIP	
2	FTFPPFPPIGP	
2	WVNRRIP	
1	SWNIDWEYYGLSFPLSP	
1	TAFDYTVP	
1	FEFLGLRLP	
1	TKYGYMFGTP	
1	IYNGDGQPYFTLTAFYP	
1	IWNQQKGRWEYIFATP	

### Phylogenetical Analyses

A phylogenetic analysis was made based on concatenated PirA and PirG protease homologs from known cyanobactin gene clusters. The maximum likelihood tree is well resolved and clearly shows the divergence between microcyclamide and piricyclamide cyanobactin gene clusters. Interestingly piricyclamide biosynthetic machinery is most similar to trichamide, anacyclamide and prenylagaramide gene clusters (Figure 2).

## Discussion

*Microcystis* is a common cyanobacterium found in freshwater lakes, ponds and reservoirs worldwide. It can form thick scums on the surface of these water bodies during the summer and early autumn. It is associated with human and livestock poisoning through the production of microcystins which are potent inhibitors of eukaryotic protein phosphatases. However, *Microcystis* strains also produce a range of other natural products [23]–[24]. Here we describe the discovery of piricyclamides from strains of the bloom-forming genus *Microcystis*. These cyclic peptides belong to the ever-expanding family of cyanobactins [4], [7], [10], [12], [15] and vary in length, amino acid content and level of post-translational modifications. The characteristic features of piricyclamides are prenylation or geranylation, disulfide bridges, and the presence of several different precursor genes in each strain. Surprisingly, the strains exhibit incomplete post-translational modification in geranyl and prenyl attachments and piricyclamides which were not prenylated or geranylated were also found to an appreciable level in some strains. This is in contrast to other cyanobactin posttranslational modifications which seem to go to completion such as heterocyclization [9]–[10]. Therefore each strain can, through genetic and chemical variation, synthesize a range of piricyclamides greater in number than might be expected from the precursor gene sequence alone. In natural systems *Microcystis* populations may synthesize a diverse selection of piricyclamide precursor peptides, which is parlayed into a myriad of piricyclamides through partial posttranslational modification.

### Disulfide Bridges

Just a small number of cyanobactins with sulphur bridges have been described so far including ulithiacyclamides, which are octapeptides as patellamides and produced by *Prochloron* [25]–[29]. In piricyclamides with disulfide bridges the length of the core peptide is not restricted to eight amino acids as in ulithiacyclamides. We found piricyclamide precursors with a double cysteine pattern and lengths varying from 12 to 22 amino acids. Piricyclamides with the disulfide bridges resemble the plant cyclotides [30]. In cyclotides the disulfide bridges stabilize the structure in addition to the cyclic structure and make the structure even more stabile [30].

### Prenylation

Some of the piricyclamides were prenylated. The most similar cyanobactins to piricyclamides are anacyclamides, planktocyclin and prenylagaramides described in the strains of *Anabaena P. rubescens* and *P. agardhii* respectively [7], [12], [31]. Interestingly, the producers of anacyclamides, prenylagaramides, and piricyclamides are all ecologically important cyanobacteria which can form toxic cyanobacterial blooms in aquatic environments.

The undecapeptides kawaguchipeptin A and antibacterial tryptophan prenylated variant kawaguchipeptin B, are found from *Microcystis aeruginosa* NIES 88 [1], [2]. Interestingly the structure of kawaguchipeptin suggests the possibility of similar type of biosynthesis to piricyclamides and other cyanobactins [4]. However, precursor genes could not be amplified from the genomic DNA of *M. aeruginosa* NIES 88 in the screening of 74 strains of *Microcystis* with the primers and PCR conditions we used for the precursor amplification. It is possible that kawaguchipeptins are made by a different type of cyanobactin pathway differing from both microcyclamide and piricyclamide or perhaps is produced by a different mechanism entirely.

The piricyclamide precursor peptides are similar to anacyclamide precursors in the strains of *Anabaena* and prenylagaramide precursors in *Planktothrix agardhii* NIES596 [7]. In piricyclamides, anacyclamides and prenylagaramides the amino acid content, peptide length and also the level of post-translational prenylation or geranylation varies. Prenylation was recently shown to be achieved by LynF in lyngyabactin biosynthesis [11]. However, LynF homologs are encoded in almost every cyanobactin gene cluster yet only some of the gene clusters produce prenylated cyanobactins. The PirF, the likely enzyme to geranylate the piricyclamides, is similar to putative cyanobactin prenylating enzymes and also to the homologues with no prenylation reported. PirF is also very similar to AcyF encoded in anacyclamide gene cluster in *Anabaena* sp. 90, which product anacyclamide was not found to have isoprenoids linkages [12]. It is also intriguing why only two of the four piricyclamides produced by *M. aeruginosa* PCC7005 are geranylated and the two others are not. It may be that the disulfide bridge prevents geranylation while the substrate amino acid is absent in the other precursor peptide. The substrate specificity for different cyclic precursors with free phenolic hydroxyl group was shown to be broad to the putative cyanobactin prenyltransferase *in vitro* [11].

Prenylation, geranylation and double prenylation was detected from the *Anabaena* strains which produced anacyclamides [12] and here similarly from the *Microcystis* which produced piricyclamides (Table 1, Table S4). This is an analogous phenomenon in eukaryotes where 3 different kinds of prenylations have been detected from proteins including farnesylation, geranylgeranylation and double geranylgeranylation [32]. The functional significance of these different types of protein prenylations in cyanobactins is unknown.

### Piricyclamides in *M. aeruginosa* PCC7005

*M. aeruginosa* PCC7005 produced piricyclamides from four precursor genes *pirE1-pirE4* as in the cyanobactin screening we found four precursor genes and piricyclamides corresponding these genes from the cell extract by LC-MS. The gene cluster was amplified and cloned into *E. coli* from *pirE2* to *pirG* (Figure 1). In the heterologous host piricyclamide with disulfide bridge from *pirE4* precursor was detected with LC-MS. The cloned fragment had *pirE3* and partial *pirE2* lacking their promoter and therefore only *pirE4* was expressed. The location of the *pirE1* precursor remained unsolved but its products were found in the *M. aeruginosa* PCC7005 cell extract. The *pirE1* gene might be located further distance away from the main cluster or in another part of the genome.

### *Microcystis* Cyanobactins

In addition to piricyclamides *Microcystis* strains also produce another cyanobactin family microcyclamides [14]. The phylogenetic analysis of the cyanobactin biosynthetic genes shows the divergence of microcyclamides and piricyclamides (Figure 2). There are many differences in these two systems, which explain the distant relationship of these cyanobactins even though produced by the same organism. Piricyclamides show greater diversity of amino acid content and number than microcyclamides. In the microcyclamides the structure is fixed to six amino acids and those usually contain heterocyclized amino acids on alternate positions with unmodified ones [3], [15], [16]–[18]. Instead of the heterocycles piricyclamides can have isoprenoid attachments and disulfide bridges. The absence of heterocycles is explained by the lack of the heterocyclase enzyme in the piricyclamide biosynthetic machinery. The difference of piricyclamides and microcyclamides is seen also in the genetic level as the biosynthetic genes of piricyclamides resemble more anacyclamide biosynthetic genes in *Anabaena* [12] than microcyclamide genes of *Microcystis* [15].

The structure of the peptide precursors is different in microcyclamides and piricyclamides. As generally in cyanobactins the microcyclamide core sequence diversity results from one precursor peptide which has several core sequences [15], [33]. In the piricyclamides each core sequence is encoded in distinct precursor gene. The different core sequences are derived from several precursors. Hence, the diversity of cyanobactins can be created from one or several precursors. The pathways with several precursors appear to allow more length variation of the core sequence as the single precursor pathway only encodes cyanobactins of the same number of amino acids. These two cyanobactin families of the genus *Microcystis* are clearly different.

Cyanobactins are similar to other naturally occurring circular proteins in bacteria, plants, fungi and animals. The cyclic nature of the peptides provides a stabile structure which is protected against proteases therefore cyclic peptides might have potential as drugs. A common role of circular proteins appears to be in the defense system of the producer organism [30]. The biological role of cyanobactins remains so far unexplained.

### Genetic Rearrangements

The *M. aeruginosa* NIES843 piricyclamide gene cluster is fragmented and inactive due to insertion sequence mediated rearrangements (Figure 1). Insertion sequences are common in the genome of *M. aeruginosa* NIES843 [19] and PCC7806 [34] and the plasticity of the genome has been suggested because of the high number of mobile elements [19], [34]. Insertion sequence mediated inactivation of gene clusters in *Microcystis* is frequently occurring event [19], [34]–[35]. In addition to inactivation of *pirA* in *M. aeruginosa* NIES843 the sequencing of the *pirE* precursors from NIES102 demonstrated that the precursors had been also target to genome re-organizations, in this case through insertion of a non-autonomous MITE element. These shorter insertion elements are also common in *Microcystis* [19] and have been associated with inactivation of the microcystin synthetase gene cluster in *Anabaena* [36]. This is even more likely to occur in other parts of the gene cluster since the precursors are only 150 bp. Interruptions in the cyanobactin genes may inactivate piricyclamide gene clusters which might be the reason why some of the predicted piricyclamides could not be detected. In addition the peptides might have other post-translational modifications, which remained to be identified.

### Conclusion

The number of cyanobactins reported is rapidly growing and the new genome sequences accelerate the discovery of new natural products. Here we describe a new group of ribosomal natural products in globally significant toxic cyanobacterium discovery driven by analysis of inactive gene cluster. Piricyclamides are cyclic peptides with a core of varying in length, amino acid content and occasionally with disulfide bridge and isoprenoid attachments such as prenyl and geranyl. We also demonstrated that the diversity of piricyclamides occurring in nature is even higher than that observed in laboratory strains. This shows the seemingly endless variation on a common theme in natural products in nature even in a single group of cyanobactins.

## Experimental Procedures

### Cyanobacterial Strains and Cultivation

The cyanobacterial strains used in the study were grown in 20–40 ml of Z8 media [37] in continuous light of 5–12 µmol m^−2 ^s^−1^ photon irradiance at 20–25°C for 7–42 days. The strains are from the University of Helsinki culture collection, except the *M. aeruginosa* PCC7005 was obtained from the PCC culture collection and *M. aeruginosa* NIES843 from NIES culture collection. Stable isotope labeling was used to determine the nitrogen and sulphur content of the cyanobactins using LC-MS. In sulphur labeling experiments the MgSO_4_ × 7 H_2_O of the Z8 media was replaced with a stable isotope of MgSO_4_ (90 atom% ^34^S; Icon). In nitrogen labeling experiments all the nitrogen sources of Z8 media was replaced with a stable isotope of NaNO_3_ (98 atom % ^15^N; Isotec™).

### Annotation of the *pir* Cluster in NIES843

The NIES843 cyanobactin gene cluster (AP009552) was re-annotated manually using Artemis (Sanger Institute). We identified three precursor genes *pirE1*-*pirE3*. The *pirE3* precursor gene was annotated in the genome as an unknown protein (MAE_00670) and identified as a precursor gene in a previous study [7]. We identified two more precursor genes, *pirE1* and *pirE2,* by searching for conserved N-terminal KKNxxPxxxxPVxR motif and C-terminal FAGD motifs in all possible open reading frames predicted using the glimmer program implemented in Artemis. This motif is present in the N-terminal leader of cyanobactin precursors as shown previously [12]. BLASTp searches were made to characterize the functions of the putative proteins in the gene cluster. The *pirA* and *pirG* genes had undergone genetic rearrangements in *M. aeruginosa* NIES843 rendering the gene cluster non-functional [7] and no cyanobactins could be identified from this strain. We screened 74 *Microcystis* strains by PCR and LC-MS in order to identify a functional cyanobactin cluster.

### DNA Extraction, PCR Amplification and Sequencing

*Microcystis* cells were collected from 20 ml of liquid culture by centrifugation at 7000 × g for 7 min (Eppendorf Centrifuge 5804R, Eppendorf). DNA was extracted using the E.Z.N.A. plant DNA mini kit (Omega Bio-Tek, Doraville, GA). The cells were shaken with glass beads (Acid Washed, 425–600 and 710–1180 microns (1∶1) Sigma-Aldrich) St. Louis, MO, USA) in a FastPrep™ cell instrument with the speed of 5 m s^−1^ for 30 s (FP120, Bio101, Thermo Electron Corp. Qbiogene, Inc) in lysis buffer with RNAase A (100 mg ml^−1^) Oligonucleotide primers kgpF1 and kgpR were designed to anneal to the conserved leader sequence and C-terminal splicing site of the NIES843 precursor genes (*pirE1-pirE3*). Genomic DNA of 74 *Microcystis* strains was screened by PCR using the kgpF1 and kgpR primers. All the primers used in the study are listed in the supporting information (Table S2). The PCR was carried out as previously described [38] except the primer concentration of 0.5 µM was used. The amplified precursor genes were cloned into the pCR 2.1-TOPO vector using a TOPO TA cloning kit (Invitrogen) according to the manufacturer’s instructions. The resulting transformants were analyzed by amplifying the insert with M13 primers and sequenced. In sequencing BigDye® Terminator Cycle Sequencing Ready Reaction kit (version 3.1) (PE Applied Biosystems) was used and the reactions were analyzed by ABI PRISM 310 Genetic Analyzer capillary gel electrophoresis (PE Applied Biosystems). All the sequences obtained in this study are deposited in GenBank (JX129189-JX129192, JQ951924).

### The *pir* Gene Cluster in PCC7005

The *pir* gene cluster was amplified from the genomic DNA of PCC7005 with primer willaF1 and luckyR in three 50 µl reaction mixtures containing 1 × PCR Buffer for Super *Taq* Plus (HT Biotechnology Ltd) 200 µmol of each nucleotide (Thermo Fischer Scientific Inc. 0.75 µmol of each primer, 0.8 U of Super *Taq* Plus proofreading polymerase (HT Biotechnology Ltd) and approximately 100 ng of template DNA. The cycling conditions were 94°C 3 min, followed by 35 cycles of 94°C for 30 s, 60°C for 30 s and 68°C for 11 min and final extension of 20 min at 68°C. A gel extraction of the PCR product was carried out as previously described [12]. The 9290 bp fragment was cloned into the pCR 2.1-TOPO vector using a TOPO TA cloning kit (Invitrogen) with insert-to-vector molar ratio of 3∶1. The vector was used to transform chemically competent *Escherichia coli* One Shot TOP10 cells according to manufacturer’s instructions. The resulting plasmids were analyzed with restriction digest with BamHI (Promega) according to manufacturer’s instructions. Plasmids with 9290 bp insert were end sequenced to confirm it contained the correct insert sequence. The entire insert was sequenced by primer walking at Beijing Genomic Institute (Beijing, China). The cyanobactin gene cluster of PCC7005 was manually (Acc. number) annotated using Artemis (Sanger Institute). The predictions for the starting sites of the proteins were checked. Two transformants carrying plasmids with the 9290 bp insert, named pPIR7005, were grown with shaking 120 rpm overnight at 28°C in 50 ml of LB medium containing 50 µg ml^−1^ of kanamycin sulphate salt (Sigma-Aldrich) for the LC-MS analysis.

### Cell Extractions for the Chemical Analysis

The *E. coli* pPIR7005 transformants and cells of *Microcystis* cultivations were collected by centrifugation at 7000 × g for 7 min. The cells were freeze dried with Supermodulyo (Edwards High Vacuum International). The freeze dried cyanobacterial cells yielded 4–10 mg dry weight and *E. coli* p7PIR7005 40 mg. The freeze dried cells were extracted with 1 ml of methanol (HiperSolv for HPLC, BDH Laboratory Supplies) and glass beads (Cell disruption medium; 0.5 mm-diameter glass beads; Scientific Industries Inc). The extracts were homogenized by shaking with FastPrep cell disrupter instrument for 30 s with the speed of 6.5 m^−1 ^s^−1^. This procedure was repeated three times and the cell extracts were kept on ice between treatments. The resulting mixture was centrifuged at 20 000 × g for 10 min and the supernatant was used in the chemical analysis.

### Chemical Analyses

The extracts were analyzed with high-performance liquid chromatography (HPLC) combined with a mass spectrometer (MS) (Agilent 1100 series LC/MSD with Ion trap XCT Plus and electrospray ion source). Peptides were separated with HPLC using a Phenomenex C_18_ (alternatively C_8_) column (2.0 mm × 150 mm; particle size, 5 µm). The mobile phase consisted of 0.1% aqueous (Milli-Q Plus purified water) formic acid (50% solution in water; Fluka, Sigma-Aldrich) as solvent A and 0.1% formic acid in isopropyl alcohol (Sigma-Aldrich) as solvent B. The percentage of solvent B was increased from 5% to 50% in 60 min. A flow rate of 0.15 ml min^−1^ was used and the column was 40°C during separation. Positive-ion mode of electrospray ionization was used. The pressure of the nebulizer gas (N_2_) was 30 lb/in^2^, the drying gas flow rate was 8 l min^−1^ and temperature was 350°C. The capillary voltage was 5000 V and the capillary offset value was 300 V. A skimmer potential of 85 V and a trap drive value of 144 were used. Spectra were recorded with a scan range of *m/z* 300 to *m/z* 2200. Piricyclamide candidates with an m/z above 2000 cannot be easily detected with the mass spectrometer used. The identification of piricyclamides was based on the ion mass predicted from the precursor amino acid sequences and the product ion spectra (MS^n^, *n = *1 to 3). The number of sulphur or nitrogen atoms in the peptides was verified with MS analysis of ^34^ S and ^15^N labelled *Microcystis* cell extracts.

### Disulfide Bridges

The presence of disulfide bridges was confirmed with a carboxyamidomethylation method [39] in which the disulfide bridge is first reduced (mass increase of 2 × 1 Da) and then free thiols are alkylated with iodoacetamide (mass increase of 2 × 57 Da). Total mass increase of 116 Da demonstrates the presence of a disulfide bridge in the molecule. One part of water and one part of dichloromethane (Sigma-Aldrich) was added to the raw methanol extract made from the cyanobacterial cells as described above. The solution was mixed and the centrifuged 10 000 × g for 5 minutes. The upper layer was removed to a 2 ml tube and evaporated and the resultant residue was used in a derivatization reaction. Derivatization was carried out as described [39] and the chemical analysis of the derivatized peptide was performed with LC-MS as described above. Matrix-assisted laser desorption/ionization time of flight (MALDI-TOF) mass spectrometric analysis was made with an Ultraflex TOF/TOF instrument (Bruker-Daltonik GmbH, Bremen, Germany). Angiotensin II (MH+ m/z 1046.542) was used as an internal standard.

### Phylogenetic Analyses

In order to explore the relationship between piricyclamide and other cyanobactin gene clusters we constructed phylogenetic trees based on a concatenated alignment of PirA (738 aa) and PirG (237 aa) homologs from cyanobactin gene clusters with known products (Table S3). A manual alignment was constructed and ambiguous and missing regions were excluded. The “oxidase” domain found in PatG was omitted from the analysis as this domain is absent from piricyclamide, anacyclamide, trichamide and prenylagaramide proteases. A total of 975 amino acids were subjected to maximum-likelihood analysis using the PHYLIP package [40]. An unrooted maximum-likelihood tree was constructed using ProtML with the JTT-F model of amino acid substitution 10 random sequence addition searches with global rearrangements. One thousand likelihood bootstrap replicates were performed under a JTT and uniform rate model with 5 random sequence additions per replicate and global rearrangements.

### Natural Sample

A freeze-dried natural sample from a bloom in Lake Tuusulanjärvi on September 7^th^ 1987 was analysed. The material contained *Aphanizomenon, Anabaena* and *Microcystis* based on preliminary microscopy in 1987. The freeze-dried material has been stored at −20°C for 23 years before this analysis. DNA was extracted and the precursor peptide genes were amplified from the natural sample as described above. Clone libraries of 16S rRNA gene and the *pirE* cyanobactin precursor gene were constructed from the natural sample. The 16S rRNA gene was amplified using the 359F and 781Ra/b primer pair [41]. BLASTn searches were made with the resulting sequences. The *pirE* cyanobactin precursor genes were amplified as described above using the kgpF1 and kgpR primer pair. The resulting transformants were analyzed and sequenced with M13 primers as described above.

## Supporting Information

Figure S1 **MS and MS2 spectra of native and derivatized peptides from** ***M. aeruginosa*** **strains as denoted in the figure.**
(PDF)

Figure S2 **Product ion mass spectra of prenylated peptide 1068.**
(PDF)

Figure S3 **Ion assignments and intensities of piricyclamide GTHLYTITP, prenylated peptide 1068 and nonprenylated peptide 1068 from** ***M. aeruginosa*** **SYKE864.**
(PDF)

Table S1 **The predicted proteins and their proposed functions in the piricyclamide gene cluster in** ***Microcystis aeruginosa*** **NIES843.**
(PDF)

Table S2 **The primers used in this study.**
(PDF)

Table S3 **The accession numbers of the N- and C-terminal proteases from the cyanobactin gene clusters with known products used in the phylogenetic analysis.**
(PDF)

Table S4 **The core sequences of piricyclamides in the** ***Microcystis*** **strains studied with calculated monoisotopic mass of corresponding protonated ions and detected variants.**
(PDF)

Table S5 **Most common fragment ions of the piricyclamide FAIFLLLP.**
(PDF)
