# Supplementary material for: Analysis of an Inactive Cyanobactin Biosynthetic Gene Cluster Leads to Discovery of New Natural Products from Strains of the Genus Microcystis
Source: PLoS One. 2012 Aug 27;7(8):e43002. doi: 10.1371/journal.pone.0043002 (PMC3428304; doi:10.1371/journal.pone.0043002)
Supplement: Figure S1 — MS and MS2 spectra of native and derivatized peptides from M. aeruginosa strains as denoted in the figure. (PDF) [file pone.0043002.s001.pdf]

**Figure S1.** MS and $MS^2$ spectra of native and derivatized peptides from *M. aeruginosa* strains as denoted in the figure. $MS^2$ spectra of prenylated piricyclamide ILGEGEGWNYNP (A), geranylated piricyclamide MSGVDYYNP (B), piricyclamide TFCDLATKQCYP (D), piricyclamide WILLADGTRPKNAP (E), piricyclamide FAIFLLLP and $^{15}$N-labelled FAIFLLLP (F), $^{15}$N-labelled piricyclamide ILGEGEGWNYNP (G), piricyclamide TLGCMNGTERCLGLP and carboxyamidomethylated TLGCMNGTERCLGLP (H), $^{15}$N-labelled piricyclamide NEFMQTGSYSGP (I), $^{15}$N-labelled piricyclamide MSGVDYYNP (J) , piricyclamide SQWGWRGLSDP and $^{15}$N-labelled SQWGWRGLSDP (K), piricyclamide, GTHLYTITP and $^{15}$N-labelled GTHLYTITP (L) and MS spectrum of geranylated piricyclamide NEFMQTGSYSGP (C).

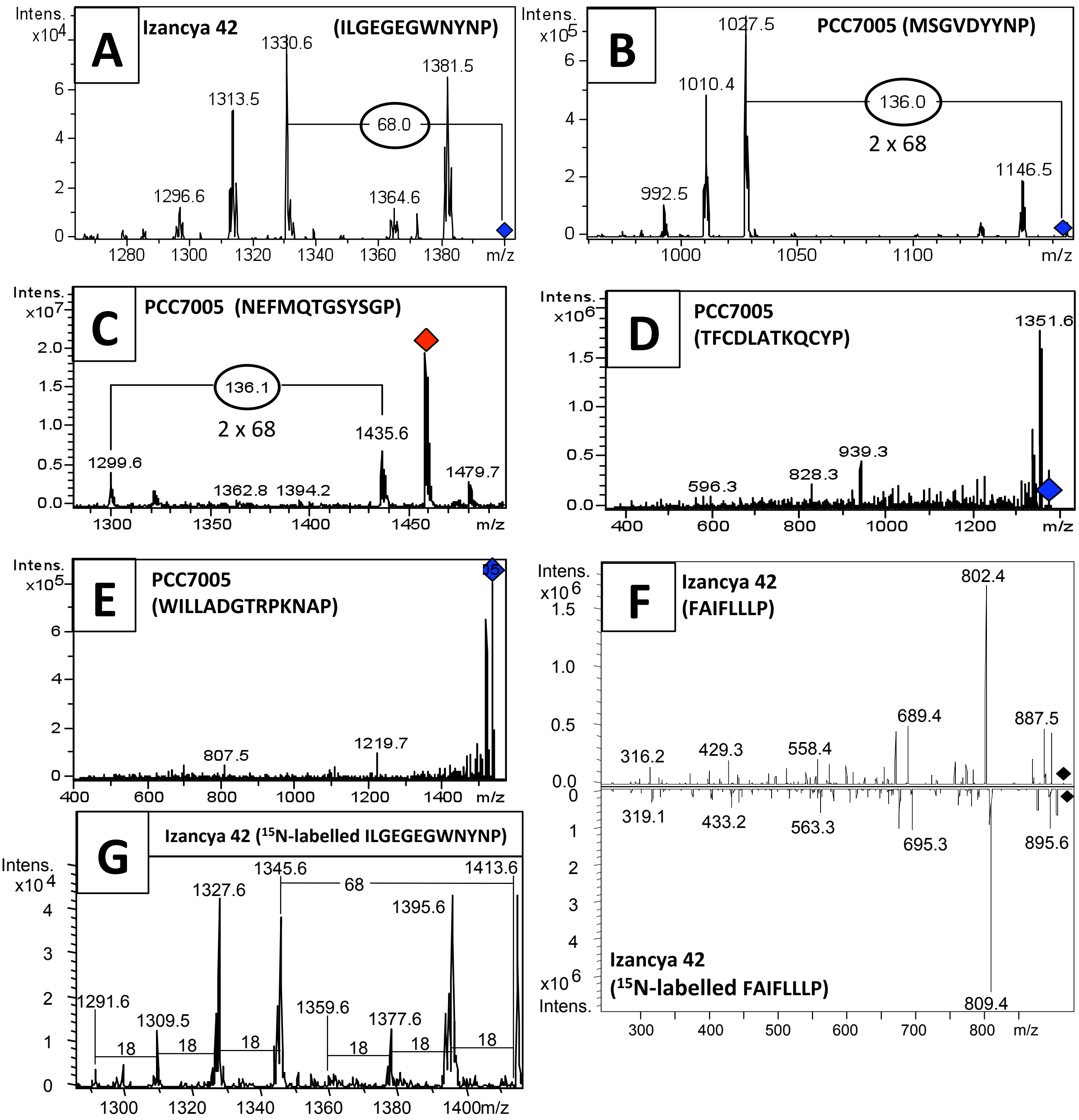

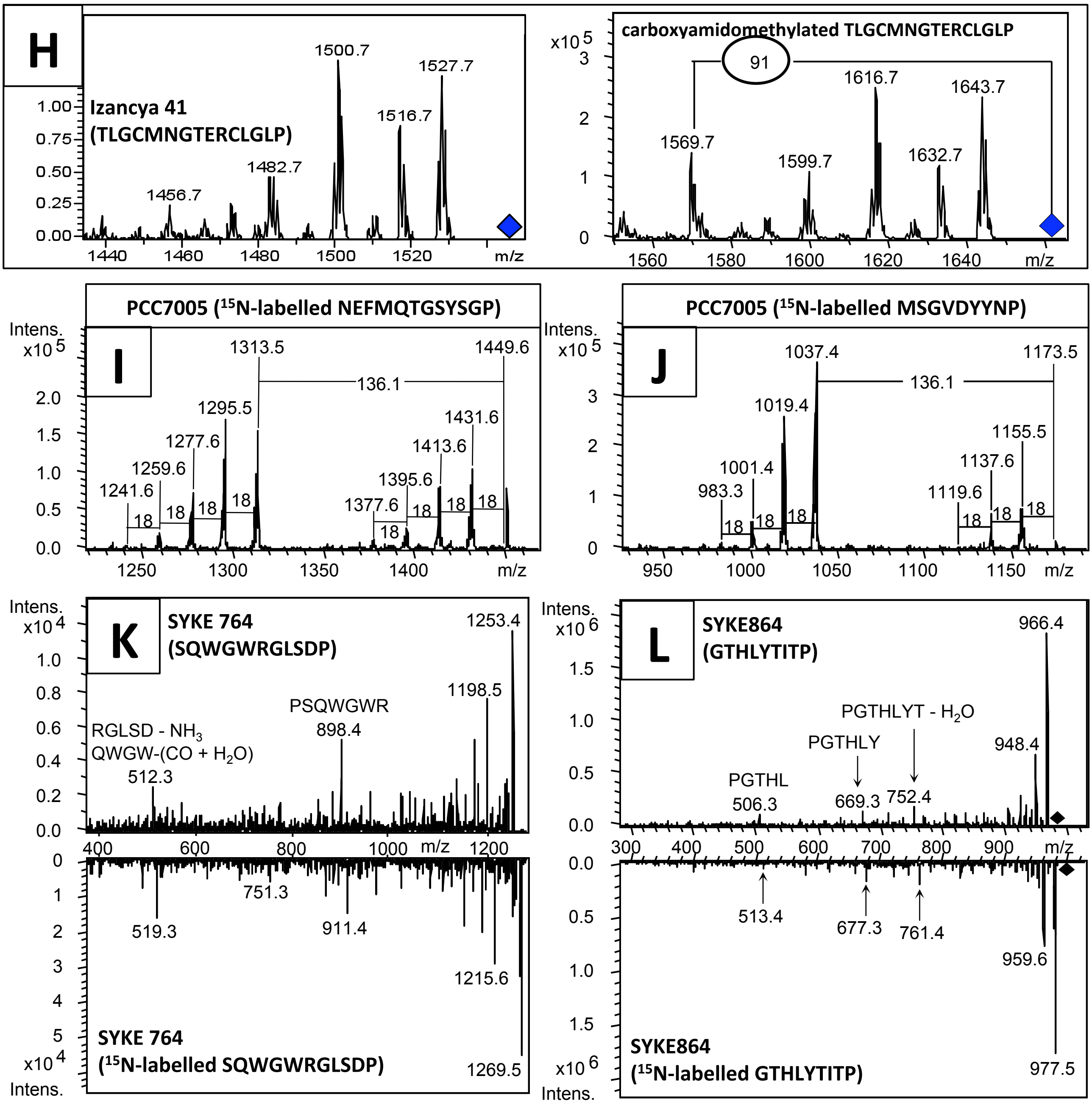
H
Izancya 41
(TLGCMNGTERCLGLP)
1456.7
1482.7
1500.7
1516.7
1527.7
m/z
carboxyamidomethylated TLGCMNGTERCLGLP
91
1569.7
1599.7
1616.7
1632.7
1643.7
m/z
PCC7005 (15N-labelled NEFMQTGSYSGP)
I
Intens.
1241.6
1259.6
1277.6
1295.5
1313.5
136.1
1377.6
1395.6
1413.6
1431.6
1449.6
18
m/z
PCC7005 (15N-labelled MSGVDYYNP)
J
Intens.
983.3
1001.4
1019.4
1037.4
136.1
1119.6
1137.6
1155.5
1173.5
18
m/z
K
SYKE 764
(SQWGWRGLSDP)
Intens.
RGLSD - NH3
QWGW-(CO + H2O)
512.3
PSQWGWR
898.4
1198.5
1253.4
m/z
519.3
751.3
911.4
1215.6
1269.5
SYKE 764
(15N-labelled SQWGWRGLSDP)
Intens.
L
SYKE864
(GTHLYTITP)
Intens.
PGTHL
506.3
PGTHLY
669.3
PGTHLYT - H2O
752.4
948.4
966.4
m/z
513.4
677.3
761.4
959.6
977.5
SYKE864
(15N-labelled GTHLYTITP)
Intens.
