# Supplementary material for: Analysis of an Inactive Cyanobactin Biosynthetic Gene Cluster Leads to Discovery of New Natural Products from Strains of the Genus Microcystis
Source: PLoS One. 2012 Aug 27;7(8):e43002. doi: 10.1371/journal.pone.0043002 (PMC3428304; doi:10.1371/journal.pone.0043002)

Figure S2. Product ion mass spectra of prenylated peptide 1068 (A), peptide 1068 (B), ^{15}N-labelled piricyclamide prenylated peptide 1068 (C), piricyclamide GTHLYTITP (D) and ^{15}N-labelled piricyclamide GTHLYTITP (E) from *M. aeruginosa* SYKE864.

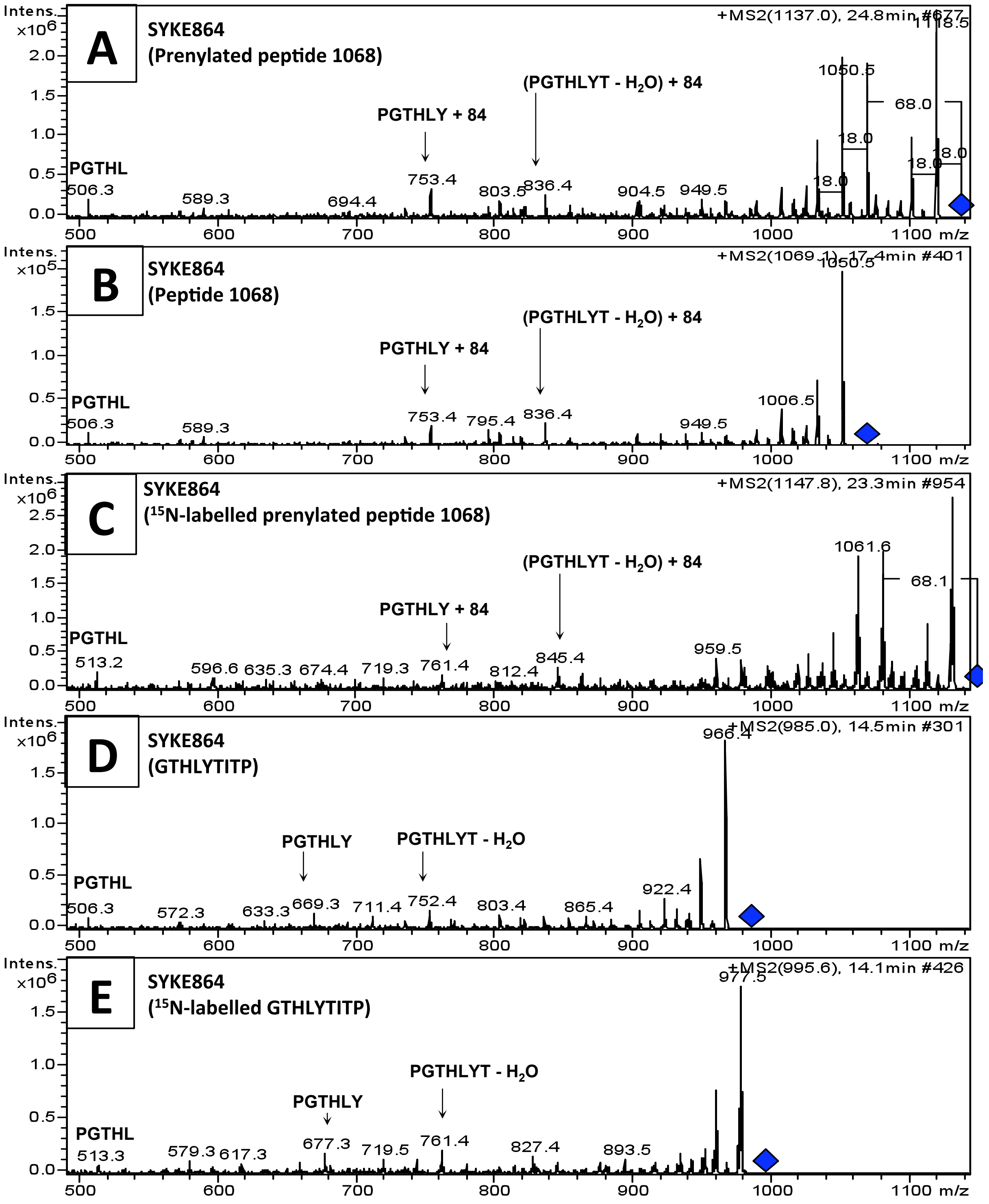

Supplement: Figure S2 — Product ion mass spectra of prenylated peptide 1068. (PDF) [file pone.0043002.s002.pdf]
