# Supplementary material for: Analysis of an Inactive Cyanobactin Biosynthetic Gene Cluster Leads to Discovery of New Natural Products from Strains of the Genus Microcystis
Source: PLoS One. 2012 Aug 27;7(8):e43002. doi: 10.1371/journal.pone.0043002 (PMC3428304; doi:10.1371/journal.pone.0043002)

Figure S3. Ion assignments and intensities of piricyclamide GTHLYTITP, prenylated peptide 1068 and nonprenylated peptide 1068 from *M. aeruginosa* SYKE864. Peptide 1068 is 84 Da larger than piricyclamide GTHLYTITP. In the ion assignments tyrosine, the 84 Da larger amino acid or a tyrosine derivative is indicated as red.

MH^+	Ion assignment	Relative intensity-% GTHLYTITP	Relative intensity-% Pr-peptide 1068	Relative intensity-% Peptide 1068	Arrow symbols
334	THL - H_2O	1,40	1,01	2,56	⟶
375	PGTH - H_2O	1,10	0,94	1,55	⟶
393	PGTH	1,86	1,80	1,71	⟶
488	PGTHL - H_2O	1,12	3,15	2,58	⟶
360	LYT - H_2O, YTI - H_2O	1,96	0,00	0,11	- - - -→
444	YTIT - (NH_3 + H_2O), (LYT+84) - H_2O, (YTI+84) - H_2O	1,36	2,49	1,78	- - - -→
396	TITP - NH_3, HLY - H_2O	2,21	1,31	1,31	— · · →
480	(HLY + 84) - H_2O	0,00	2,02	2,43	— · · →
497	THLY - H_2O, HLYT - H_2O	1,95	0,17	0,43	
581	(THLY+84) - H_2O, (HLYT+84) - H_2O	0,21	2,43	2,17	

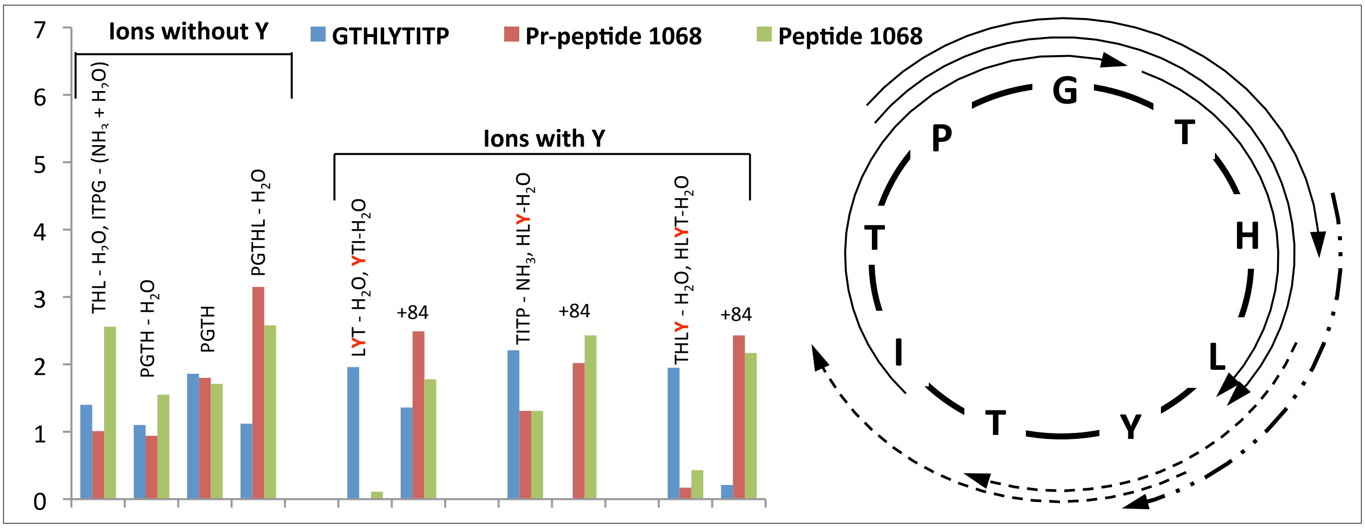

Supplement: Figure S3 — Ion assignments and intensities of piricyclamide GTHLYTITP, prenylated peptide 1068 and nonprenylated peptide 1068 from M. aeruginosa SYKE864. (PDF) [file pone.0043002.s003.pdf]
