# Supplementary material for: Analysis of an Inactive Cyanobactin Biosynthetic Gene Cluster Leads to Discovery of New Natural Products from Strains of the Genus Microcystis
Source: PLoS One. 2012 Aug 27;7(8):e43002. doi: 10.1371/journal.pone.0043002 (PMC3428304; doi:10.1371/journal.pone.0043002)
Supplement: Table S1 — The predicted proteins and their proposed functions in the piricyclamide gene cluster in Microcystis aeruginosa NIES843. (PDF) [file pone.0043002.s004.pdf]

Table S1. The predicted proteins and their proposed functions in the piricyclamide gene cluster in *Microcystis aeruginosa* NIES843.

Protein	Accession number	Length (aa)	Predicted function	Blast result	
				Identity (%)	Organism
PirE1		61	Precursor	33	*Anabaena* BIR260
	MAE_00430	63	Unknown		
	MAE_00440	94	Hypothetical		
	MAE_00450	98	Hypothetical		
	MAE_00460	73	Unknown		
PirE2		51	Precursor	44	*Anabaena* PH256
	MAE_00470	66	Unknown		
	MAE_00480	130	Hypothetical		
	MAE_00490	147	HNH endonuclease		
PirF	MAE_00500	298	Prenylation	72	*Anabaena* 90
	MAE_510	110	Hypothetical		
	MAE_520	222	Hypothetical		
	MAE_530	148	Hypothetical		
	MAE_540	189	Hypothetical		
PirA	MAE_00550	123	C-terminal protease (1/3)	65	
	MAE_560	552	Transposase		
	MAE_570	495	Hypothetical		
	MAE_580	62	Hypothetical		
PirA	MAE_00590	304	C-terminal protease (2/3)		
	MAE_00600	49	Hypothetical		
	MAE_00610	67	Unknown		
	MAE_00620	53	Unknown		
	MAE_00630	371	Transposase		
PirA	MAE_00640	356	C-terminal protease (3/3)		
PirB	MAE_00650	69	Associated with cyanobactin biosynthesis	62	*Anabaena* 90
PirC	MAE_00660	319	Associated with cyanobactin biosynthesis (75/319)	80	*M. aeruginosa* PCC7806
PirE3	MAE_00670	51	Precursor	36	*Anabaena* 1tu39s8
	MAE_00680	260	Hypothetical		
PirG	MAE_00690 (1/2)	169	N-terminal protease, macrocyclaze (1/2)	58	*T. erythraeum* IMS101
PirG	MAE_00700	483	N-terminal protease, macrocyclaze	61	*T. erythraeum*
