# Supplementary material for: Analysis of an Inactive Cyanobactin Biosynthetic Gene Cluster Leads to Discovery of New Natural Products from Strains of the Genus Microcystis
Source: PLoS One. 2012 Aug 27;7(8):e43002. doi: 10.1371/journal.pone.0043002 (PMC3428304; doi:10.1371/journal.pone.0043002)
Supplement: Table S2 — The primers used in this study. (PDF) [file pone.0043002.s005.pdf]

Table S2. The primers used in this study.

Primer	Sequence 5’-3’
kgpF1	atgaaaaccaaaaaactgacacccc
kgpR	gcgggagacgacgcagagtaa
luckyR	ctatttagaaacagcccaagaacgcac
willaF	ctcaatggattctgttggccg
willaR	cggccaacagaatccattgag
luckyF	ccggtatccgaaagccacgaattaac
msgF	tacaggtctgacaccctccatgt
msgR	acatggagggtgtcagacctgta
pirF102R	agaggataaatcggctcaacatc
p843F	cataacaattcccgaagagc
