# Supplementary material for: Analysis of an Inactive Cyanobactin Biosynthetic Gene Cluster Leads to Discovery of New Natural Products from Strains of the Genus Microcystis
Source: PLoS One. 2012 Aug 27;7(8):e43002. doi: 10.1371/journal.pone.0043002 (PMC3428304; doi:10.1371/journal.pone.0043002)
Supplement: Table S3 — The accession numbers of the N- and C-terminal proteases from the cyanobactin gene clusters with known products used in the phylogenetic analysis. (PDF) [file pone.0043002.s006.pdf]

Table S3. The accession numbers of the N- and C-terminal proteases from the cyanobactin gene clusters with known products used in the phylogenetic analysis.

Strain	Accession number		
	N-terminal protease PirA-like	C-terminal protease PirG-like	Cyanobactin
M. aeruginsa PCC7005	AFK79993	AFK79998	Piricyclamide
T. erythraeum IMS101	YP_722055	YP_722058	Trichamide
Anabaena 90	ACK37888	ADA00395	Anacyclamide
P. agardhii NIES596	AED99426	AED99446	Prenylagaramide
Prochloron (uncultured)	ACA04487	ACA04494	Trunkamide, patellin
Prochloron (uncultured)	AAY21150	AAY21156	Patellamide, ulithiacyclamide
Nostoc TAU strain IL-184-6	ACA04480	ACA04486	Tenuecyclamide
M. aeruginosa PCC7806	CAP64335	CAP64342	Microcyclamide
M. aeruginosa NIES298	CAO82081	CAO82089	Microcyclamide
Arthrospira platensis NIES39	BAI93369	BAI93347	Arthrospiramide
Lyngbya PCC8106	ZP_01623699	ZP_01623710	Lyngbyabactin
