# Supplementary material for: Analysis of an Inactive Cyanobactin Biosynthetic Gene Cluster Leads to Discovery of New Natural Products from Strains of the Genus Microcystis
Source: PLoS One. 2012 Aug 27;7(8):e43002. doi: 10.1371/journal.pone.0043002 (PMC3428304; doi:10.1371/journal.pone.0043002)
Supplement: Table S4 — The core sequences of piricyclamides in the Microcystis strains studied with calculated monoisotopic mass of corresponding protonated ions and detected variants. (PDF) [file pone.0043002.s007.pdf]

Table S4. The core sequences of piricyclamides in the *Microcystis* strains studied with calculated monoisotopic mass of corresponding protonated ions and detected variants. Number of nitrogen and sulphur atoms solved from ^{15}N- and ^{34}S-labelling experiments is in parenthesis.

		Monoisotopic *m/z* of $[M+H]^+$							
		Calculated	Detected						
Microcystis strain	Core sequence	Unmodified	Unmodified	Disulfide bridge	Prenylated	Geranylated	MetO	^{15}N	^{34}S
PCC7005	MSGVDYYNP	1027				1163	1179	1173 (10)	
	NEFMQTGSYSGP	1299				1435	1451	1449 (14)	
	TFCDLATKQCYP	1371		1369				1383 (14)	
	WILLADGTRPKNAP	1533	1533					1553 (20)	
Izancya 36, 41	TLGCMNGTERCLGLP	1546		1544			1560	1563 (19)	1550 (2)
	DWGTFCVQEDGEGNCKEWYEVP	2573							
Izancya 42	YSNVLPP	771							
	FAIFLLLP	915	915					923 (8)	
	ILGEGEGWNYNP	1330			1398			1413 (15)	
SYKE 764	SQWGWRGLSDP	1270	1270					1287 (17)	
	ILGEGEGWNYNP	1330							
	GWGTFCVGEDGDGNCEEWYELP	2445							
SYKE 864	GTHLYTITP	984	984					995 (11)	
	TFCDLATKQCYP (insert)	1371							
	DWGTFCVQEDGEGNCKEWYELP	2587							
	Unknown	1068	1068		1136			1079, 1147 (11)	
NIES 843	APLWDLVRWGAP	1362							
	TFCDLATKQCYP	1371							
	DWGTFCVQEDGEGNCKEWYELP	2587							
