# Supplementary material for: Analysis of an Inactive Cyanobactin Biosynthetic Gene Cluster Leads to Discovery of New Natural Products from Strains of the Genus Microcystis
Source: PLoS One. 2012 Aug 27;7(8):e43002. doi: 10.1371/journal.pone.0043002 (PMC3428304; doi:10.1371/journal.pone.0043002)
Supplement: Table S5 — Most common fragment ions of the piricyclamide FAIFLLLP. (PDF) [file pone.0043002.s008.pdf]

Table S5. Most common fragment ions of the piricyclamide FAIFLLLP

Pepide and fragmnet sequences								Ions (m/z)		
								**$+H^+$**	**$+H^+$ - CO**	**$+H^+$ – (CO + NH_3)**
F	A	I	F	L	L	L	P	**915**	**887**	**870**
	A	I	F	L	L	L	P	**768**	740	**723**
		I	F	L	L	L	P			652
			F	L	L	L	P	**584**	556	539
				L	L	L	P		409	392
					L	L	P	324	296	
	I	F	L	L	L	P	F	**844**	816	799
		F	L	L	L	P	F	**731**	703	686
			L	L	L	P	F	**584**	556	539
				L	L	P	F	471	443	**426**
					L	P	F	**358**	330	313
	F	L	L	L	P	F	A	**802**	**774**	**757**
		L	L	L	P	F	A	**655**	**627**	**610**
			L	L	P	F	A	**542**	**514**	**497**
				L	P	F	A	**429**	**401**	384
					P	F	A	**316**	288	
	L	L	L	P	F	A	I	**768**	740	**723**
		L	L	P	F	A	I	**655**	**627**	**610**
			L	P	F	A	I	**542**	**514**	**497**
				P	F	A	I	**429**	**401**	384
					F	A	I	**332**		**287**
	L	L	P	F	A	I	F	**802**	**774**	**757**
		L	P	F	A	I	F	**689**	**661**	**644**
			P	F	A	I	F	**576**	**548**	**531**
				F	A	I	F	479	451	**434**
					A	I	F	**332**		**287**
						I	F	**261**		
	L	P	F	A	I	F	L	**802**	**774**	**757**
		P	F	A	I	F	L	**689**	**661**	**644**
			F	A	I	F	L	592		**547**
				A	I	F	L	**445**	**417**	**400**
					I	F	L	**374**		**329**
						F	L	**261**		
	P	F	A	I	F	L	L	**802**	**774**	**757**
		F	A	I	F	L	L	705		**660**
			A	I	F	L	L	**558**	**530**	**513**
				I	F	L	L	**487**	**459**	**442**
					F	L	L	**374**		**329**
	F	A	I	F	L	L	L	**818**	790	**773**
		A	I	F	L	L	L	**671**	**643**	**626**
			I	F	L	L	L	**600**	572	**555**
				F	L	L	L	**487**	**459**	**442**
					L	L	L	340		**295**

Dipeptide LP, PF, FA, AI and LL ions were unstable in ion trap

Ions with relative intensity over 1% are in boldface
